# Agreement of the 2020 Brazilian Society of Cardiology cardiac risk stratification protocol in cardiac rehabilitation programs: a cross-sectional study

**DOI:** 10.1590/1806-9282.20260217

**Published:** 2026-08-07

**Authors:** Laíza Delatorre Mardegan Borges, Jéssica Malek da Silva, Kemilly Pereira Florêncio, Maria Fernanda de Souza Moreno Lopes, Letícia Soares Alves, Maria Clara de Souza Moreno Lopes, Carlos Eduardo da Costa Nunes Bosso, Luiz Carlos Marques Vanderlei

**Affiliations:** 1 Universidade Estadual Paulista “Júlio de Mesquita Filho”, School of Technology and Sciences, Department of Physiotherapy – Presidente Prudente (SP), Brazil.; 2 Universidade do Oeste Paulista, Department of Medicine – Presidente Prudente (SP), Brazil.

**Keywords:** Exercise, Rehabilitation, Cardiac rehabilitation, Risk assessment

## Abstract

**BACKGROUND:**

Cardiac risk stratification protocols are widely used in cardiac rehabilitation programs, but divergent classifications limit their applicability. In 2020, the Brazilian Society of Cardiology updated its protocol; however, its agreement with established protocols and inter-rater reliability in cardiac rehabilitation programs remain unclear.

**OBJECTIVE:**

The aim of this study was to evaluate the agreement between the 2020 Brazilian Society of Cardiology cardiac risk stratification protocol and protocols used in clinical practice and to assess inter-rater agreement in its application.

**DESIGN AND SETTING:**

Cross-sectional observational study conducted in a cardiac rehabilitation program at a public university in Brazil.

**METHODS:**

Medical records of 51 cardiac rehabilitation programs participants were independently analyzed by two cardiovascular physical therapists using the 2020 Brazilian Society of Cardiology, American Association of Cardiovascular and Pulmonary Rehabilitation, American College of Sports Medicine, Pashkow, and 2013 Brazilian Society of Cardiology protocols. Agreement between protocols and inter-rater reliability was assessed using the Kappa coefficient.

**RESULTS:**

The 2020 Brazilian Society of Cardiology protocol showed moderate agreement with Pashkow (k=0.544; 95%CI 0.340–0.748; p<0.001) and the 2013 Brazilian Society of Cardiology protocol (k=0.708; 95%CI 0.498–0.918; p<0.001), good-to-excellent agreement with American Association of Cardiovascular and Pulmonary Rehabilitation (k=0.780; 95%CI 0.604–0.956; p<0.001), and weak, nonsignificant agreement with American College of Sports Medicine (k=0.003; 95%CI -0.015 to 0.021 p=0.866). Inter-rater analysis demonstrated excellent agreement between evaluators (k=0.843; 95%CI 0.672–1.014; p<0.001).

**CONCLUSION:**

The findings provide preliminary evidence regarding the agreement and reproducibility of the 2020 Brazilian Society of Cardiology protocol compared with established cardiac rehabilitation programs protocols; however, further studies are needed to evaluate its predictive validity for exercise-related events.

## INTRODUCTION

Exercise-based cardiac rehabilitation programs (CRPs) are class I, level A interventions for preventing and treating cardiovascular diseases, the leading cause of death worldwide^1^. Adverse events during supervised exercise, from minor clinical signs to severe complications such as acute myocardial infarction and cardiorespiratory arrest, have been reported^2,3,4,5,6^.

Risk stratification protocols classify patients as high, moderate, or low risk^7,8,9^ and are mostly based on complementary test variables associated with morbidity and mortality^10^. Despite limited exercise-specific validation, these protocols guide CRP professionals in monitoring and exercise prescription^8^.

Silva et al.^9^ identified seven protocols used in CRPs: American College of Sports Medicine (ACSM)^11^, Brazilian Society of Cardiology (BSC)^12^, Association of Cardiovascular and Pulmonary Rehabilitation (AACVPR)^13^, American Heart Association (AHA)^14^, Société Française de Cardiologie^15^, Spanish Society of Cardiology^16^, and Pashkow^17^. Classification criteria vary, complicating protocol selection and reliability^8,9,18^. Santos et al.^8^ reported low-to-moderate agreement among protocols, suggesting divergent classifications for the same patient and limiting clinical applicability^8,9,10^.

Inter-rater agreement studies indicate moderate-to-good consistency^18^, with disagreements related to hemodynamic standards, rhythm terminology, left ventricular dysfunction criteria, variability in clinical interpretation, and limited familiarity with some protocols.

## METHODS

### Study design and ethical aspects

This cross-sectional observational study was conducted in 2024, following the Strengthening the reporting of observational studies in epidemiology (STROBE) guidelines^19^. Data were obtained from the medical records of 51 CRP participants at São Paulo State University (UNESP), Presidente Prudente, Brazil. All participants provided informed consent. The study was approved by the institutional Research Ethics Board (CAAE: 78227324.8.0000.5402).

### Participants

Sample size was calculated based on a minimum expected Kappa coefficient of 0.40, according to recommendations for agreement studies^20,21^, 5% error, and 80% power, yielding a minimum sample size of 42 participants, which was increased to 47 to account for potential dropouts^20,21^. Data extracted included sex, age, body mass index (BMI), and primary clinical diagnosis. Participants without cardiovascular disease were assigned to the prevention group.

### Risk stratification

Stratification used clinical history, risk factors, signs/symptoms, and complementary/laboratory tests (most recent available), including blood glucose, lipids, and cardiovascular assessments. The 2020 BSC protocol^1^ was compared with AACVPR^13^, ACSM^11^, Pashkow^17^, and 2013 BSC^12^, selected for clinical relevance^8,9,10,22^. Two cardiovascular physical therapists independently performed stratification; participant identifiers were removed to ensure blinding. Patients were classified as low, moderate, high risk, or unstratified.

### Protocol summaries

#### 2020 BSC^1^

Stratifies risk based on time since cardiovascular event, intervention, or decompensation; functional capacity via exercise stress testing and/or ergospirometry; ischemic signs or symptoms at low workloads; heart failure or angina symptoms; additional clinical characteristics (e.g., chronic renal failure, oxygen desaturation, complex ventricular arrhythmia); and clinician judgment in pre-participation evaluation.

#### AACVPR^13^

Uses complementary tests, especially maximal exercise testing, to identify ischemic or other symptoms during exercise or recovery. Recommended for patients with a history of Acute Myocardial Infarction (AMI); absence of testing can complicate classification. Previously associated with prediction of complications during CRP.

#### ACSM^11^

Stratifies based on cardiovascular, metabolic, or respiratory risk factors and signs or symptoms; complementary test results are not required. Positive risk factors include family history of AMI, coronary revascularization, or sudden death; smoking; hypertension; dyslipidemia; fasting glucose >100 mg/dL; obesity; and inactivity. High-Density Lipoprotein Cholesterol >60 mg/dL is a negative risk factor. Signs assessed include angina-equivalent pain, dyspnea, syncope, edema, palpitations, claudication, heart murmur, and unusual fatigue.

#### Pashkow^17^

Developed for patients with a history of AMI. Uses complementary tests, including progressive exercise stress testing, electrocardiogram, and echocardiogram, to stratify risk. Known for its highest concordance with other protocols and strong inter-protocol agreement.

#### 2013 BSC^12^

Based primarily on maximal exercise testing to identify ischemia, ventricular dysfunction, arrhythmias, and conduction disorders. Classifies patients as low, moderate, or high risk, recommending reassessment every 6–12 months. Any criterion classified as moderate or high risk determines the overall risk category.

### Statistical analysis

Descriptive statistics characterized the sample (mean±standard deviation or frequencies). Normality was tested with Shapiro-Wilk. Agreement between protocols and inter-rater reliability used Kappa and their respective 95% confidence intervals (k<0.40 weak; 0.40–0.75 moderate; >0.75 excellent). Analyses were conducted in Statistical Package for the Social Sciences v22.0; significance was set at p<0.05.

## RESULTS

This study analyzed data from 51 participants enrolled in a CRP, with no losses during the risk stratification process. As shown in Table 1, the sample comprised older adults of both sexes, with a mean age of 69.37±10.82 years and a mean BMI of 27.85±4.08 kg/m^2^, indicating an overweight status. Coronary insufficiency and AMI were the most prevalent diagnoses, observed in 19 and 11 participants, respectively.

**Table 1:** Sample characterization and complementary and laboratory tests (n=51).

Characteristics	Values
Sex n (%)	
Male	25 (49.01)
Female	26 (50.98)
Age (years)	69.37±10.82
BMI (kg/m^2^)	27.85±4.08
Principal diagnosis n (%)	
Coronary insufficiency	19 (37.25)
AMI	11 (21.57)
Prevention	9 (17.65)
Cardiac arrhythmias	3 (5.88)
Heart failure	3 (5.88)
Dilated cardiomyopathy	3 (5.88)
Others	3 (5.88)
Exam n (%)	
Exercise stress test	37 (72.55)
Echocardiography	32 (62.75)
Coronary artery angiography	23 (45.09)
Laboratory exam	21 (41.18)
Electrocardiogram	19 (37.25)
Holter monitoring	11 (21.57)
Doppler echocardiogram	11 (21.57)
Ultrasound	8 (15.69)
Myocardial scintigraphy	6 (11.76)
ABPM	4 (7.84)
Angiotomography	3 (5.88)

Values are presented as mean±standard deviation or n (%). n: number of participants; %: percentage; BMI: body mass index (kg/m^2^); AMI: acute myocardial infarction; ABPM: ambulatory blood pressure monitoring.

Medical records included several complementary and laboratory examinations (Table 1), with exercise stress tests and echocardiograms being the most frequent, reported in 37 and 32 records, respectively. Table 2 presents the risk classification distribution for each protocol and the number of unstratified participants.

**Table 2:** Patient risk classification by analyzed protocols and agreement with the 2020 Brazilian Society of Cardiology protocol.

Protocols	Low risk	Moderate risk	High risk	Unstratified
2020 BSC	39 (76.47)	7 (13.72)	1 (1.96)	4 (7.84)
AACVPR	35 (68.62)	8 (15.68)	3 (5.88)	5 (9.80)
ACSM	2 (3.92)	3 (5.88)	46 (90.19)	0 (0)
Pashkow	29 (56.86)	15 (29.41)	2 (3.92)	5 (9.80)
2013 BSC	38 (74.50)	7 (13.72)	1 (1.96)	5 (9.80)
**Protocols**	**k (95%CI)**		**p-value**	
2020 BSC vs. AACVPR	0.780 (0.604–0.956)		<0.001	
2020 BSC vs. ACSM	0.003 (-0.015 to 0.021)		0.866	
2020 BSC vs. Pashkow	0.544 (0.340–0.748)		<0.001	
2020 BSC vs. 2013 BSC	0.708 (0.498–0.918)		<0.001	

Values as n (%). AACVPR: American Association of Cardiovascular and Pulmonary Rehabilitation; ACSM: American College of Sports Medicine; BSC: Brazilian Society of Cardiology; k: kappa coefficient; 95%CI: 95% confidence interval. The ACSM protocol does not require complementary examinations for risk stratification.

Agreement between the 2020 BSC protocol and the other protocols is presented in Table 2. Significant moderate agreement was observed with the Pashkow and 2013 BSC protocols and significant excellent agreement with the AACVPR protocol. No significant agreement was found with the ACSM protocol, which showed weak concordance.

Inter-evaluator agreement for the updated BSC protocol is shown in Table 3. Three discrepancies were identified, mainly involving the interpretation of moderate-risk criteria or classification as unstratified in cases with incomplete complementary examinations. Four participants (7.84%) could not be stratified due to missing complementary examinations. Overall, the protocol demonstrated excellent and significant inter-rater agreement (κ=0.843; 95%CI 0.672–1.014; p<0.001).

**Table 3:** Inter-rater agreement in risk stratification using the 2020 Brazilian Society of Cardiology protocol.

2020 BSC	% Agreement	% Disagreement
Ev.1 vs. Ev.2	94.11 (n=48)	5.88 (n=3)
**2020 BSC**	**k (95%CI)**	**p-value**
Ev.1 vs. Ev.2	0.843 (0.672–1.014)	<0.001

Values expressed as a percentage (number of participants). BSC: Brazilian Society of Cardiology; Ev: evaluator; k: kappa coefficient; 95%CI: 95% confidence interval.

## DISCUSSION

The main findings indicate that the 2020 BSC protocol showed moderate agreement with the Pashkow and previous BSC protocols, excellent agreement with the AACVPR protocol, and weak agreement with the ACSM protocol. Additionally, the updated BSC protocol demonstrated excellent inter-rater agreement (κ=0.84), likely reflecting greater objectivity and clearer criteria than the 2013 version (κ=0.73)^18^. Despite the overall clarity of the protocol, the item “other clinical characteristics” remains partially subjective.

To our knowledge, this is the first study to assess inter-rater agreement and concordance between the 2020 BSC protocol and other cardiac risk stratification protocols in CRP participants. The 2020 BSC protocol failed to stratify 7.84% of participants, compared with 9.80% for the AACVPR, Pashkow, and 2013 BSC protocols, mainly due to missing complementary examinations^23^.

The lack of agreement between the ACSM and 2020 BSC protocols should be interpreted considering their different conceptual approaches. Unlike protocols developed for supervised cardiac rehabilitation, the ACSM protocol was designed for broader exercise screening and adopts a more conservative classification, categorizing most individuals with cardiovascular disease as high risk. Since most participants had cardiovascular disease, the predominance of high-risk classifications under the ACSM protocol was expected. Santos et al.^8^ also reported low agreement between the ACSM and other protocols.

Santos et al.^8^ reported moderate agreement between the AACVPR and the 2013 BSC protocol (k=0.52; p<0.001), whereas the present study found excellent agreement between the AACVPR and the 2020 BSC protocol (k=0.78; 95%CI 0.604–0.956; p<0.001). This finding may reflect similarities in complementary examinations and exercise-related criteria.

Inter-rater analysis demonstrated excellent agreement between evaluators. The three disagreements mainly involved the interpretation of moderate-risk criteria or classification as unstratified cases with incomplete complementary examinations. However, inter-rater reliability was assessed using only two evaluators from the same institution, which may limit external generalizability. Intra-rater reliability was not evaluated.

Despite the significant agreement observed between the 2020 BSC protocol and most evaluated protocols, except ACSM, the findings should be interpreted cautiously. This study evaluated agreement rather than predictive validity for adverse events during exercise. Previous studies^10,22^ indicate that current cardiac risk stratification protocols have limited ability to predict events during supervised exercise, and the predictive ability of the 2020 BSC protocol remains untested.

Additionally, the sample size calculation did not account for multiple pairwise comparisons or hierarchical data structure related to inter-rater assessments and unstratified cases. The single-center design and limited access to complementary examinations may restrict the external validity of the findings.

## CONCLUSION

The 2020 BSC protocol demonstrated moderate-to-excellent agreement with most evaluated cardiac risk stratification protocols and excellent inter-rater reliability. These findings contribute to the methodological assessment of the updated protocol and support its reproducibility. However, because this study did not evaluate the prediction of adverse events during exercise, no conclusions can be drawn regarding predictive validity or exercise safety. Further prospective studies are needed to evaluate the ability of the 2020 BSC protocol to predict clinical outcomes and exercise-related complications in cardiac rehabilitation programs.

## Data Availability

The datasets generated and/or analyzed during the current study are available from the corresponding author upon reasonable request.
